# Akap350 Recruits Eb1 to The Spindle Poles, Ensuring Proper Spindle Orientation and Lumen Formation in 3d Epithelial Cell Cultures

**DOI:** 10.1038/s41598-017-14241-y

**Published:** 2017-11-02

**Authors:** Evangelina Almada, Facundo M. Tonucci, Florencia Hidalgo, Anabela Ferretti, Solange Ibarra, Alejandro Pariani, Rodrigo Vena, Cristián Favre, Javier Girardini, Arlinet Kierbel, M. Cecilia Larocca

**Affiliations:** 1 Instituto de Fisiología Experimental, Consejo de Investigaciones Científicas y Técnicas (CONICET), Facultad de Ciencias Bioquímicas y Farmacéuticas, Universidad Nacional de Rosario, Rosario, Argentina; 2 Instituto de Biología Molecular y Celular de Rosario, CONICET, Facultad de Ciencias Bioquímicas y Farmacéuticas, Universidad Nacional de Rosario, Rosario, Argentina; 3 Instituto de Investigaciones Biotecnológicas Dr. Rodolfo A. Ugalde, Universidad Nacional de San Martín, CONICET, San Martín, Buenos Aires, Argentina

## Abstract

The organization of epithelial cells to form hollow organs with a single lumen requires the accurate three-dimensional arrangement of cell divisions. Mitotic spindle orientation is defined by signaling pathways that provide molecular links between specific spots at the cell cortex and astral microtubules, which have not been fully elucidated. AKAP350 is a centrosomal/Golgi scaffold protein, implicated in the regulation of microtubule dynamics. Using 3D epithelial cell cultures, we found that cells with decreased AKAP350 expression (AKAP350KD) formed polarized cysts with abnormal lumen morphology. Analysis of mitotic cells in AKAP350KD cysts indicated defective spindle alignment. We established that AKAP350 interacts with EB1, a microtubule associated protein that regulates spindle orientation, at the spindle poles. Decrease of AKAP350 expression lead to a significant reduction of EB1 levels at spindle poles and astral microtubules. Conversely, overexpression of EB1 rescued the defective spindle orientation induced by deficient AKAP350 expression. The specific delocalization of the AKAP350/EB1complex from the centrosome decreased EB1 levels at astral microtubules and lead to the formation of 3D-organotypic structures which resembled AKAP350KD cysts. We conclude that AKAP350 recruits EB1 to the spindle poles, ensuring EB1 presence at astral microtubules and proper spindle orientation during epithelial morphogenesis.

## Introduction

Epithelial cells are characterized by their multicellular organization, where the apico-basal asymmetry of each cell is coordinated with the apico-basal asymmetry of its neighbors. This synchronized cell polarity is responsible for the common function of epithelia: to generate and maintain two compartments with different composition. Most epithelial cells have a single apical pole, constituting a columnar type of epithelial polarity. The organization of these cells to form hollow organs with a single lumen requires a precise three-dimensional arrangement of cell divisions: each cell must divide symmetrically within the epithelial plane, so that both resulting daughter cells remain in the same plane. This type of cell division requires the orientation of mitotic spindles within the planar axis. A common feature of spindle orientation is the existence of signaling pathways that provide molecular links between the cell cortex and astral microtubules, thus generating dynamic forces on the spindle to define its accurate orientation [reviewed in^1^].

Studies using 3D epithelial cell cultures have significantly contributed to the understanding of different factors that provide cortical cues for symmetric epithelial cell division within the planar plane. Normal epithelial cells grown in a matrix rich in extracellular proteins form organotypic epithelial structures, where each cell organizes its apical membrane facing a unique central lumen (cysts). Cell failure to orient its mitotic spindle within the epithelia plane leads to the formation of abnormal cysts with more than one lumen^2^. Studies using 3D MDCK cell cultures showed that α3-β1 integrin activation at the basolateral membrane^3^ and activation of cdc42 and PI(3) kinase are essential for proper spindle orientation once the apico-basal axis has been established^4^. A recent study using the same model revealed that, during mitosis, the junctional adhesion molecule-A (JAM-A) activates cdc42 and simultaneously promotes PIP3 and dynactin subunit p150glued enrichment at cell adhesion junctions^5^. Despite the characterization of the role of this complex in spindle orientation, the mechanism underlying its interaction with astral microtubules has not been uncovered.

The centrosome is the major microtubule-organizing center of animal cells, responsible for providing MT nucleation sites where MT assembly is initiated. During mitosis, the interphase network of microtubules goes through intense remodeling. In this scenario, the duplicated centrosomes separate, forming two opposing MTOCs at the spindle poles, and experience a marked increase in size and nucleation capacity (centrosome maturation). Mature centrosomes organize two main arrangements of microtubules: astral microtubules, with their plus-ends exploring the cell cortex, and kinetochore fibers, with their plus-ends hitched to chromosomes [reviewed in^6^]. EB1 is a microtubule binding protein generally recognized by its capacity of directly binding to interphase microtubule plus ends, which also localizes to the centrosome, astral microtubules and kinetochore fibers^7^. Several lines of evidence indicate that EB1 participates in spindle orientation in epithelial cells. Primary studies performed in yeast characterized Bim1, the budding yeast orthologous of EB1, as a central regulator of spindle orientation^8^. Concomitantly, studies in drosophila indicated that EB1 is also a crucial factor for spindle orientation during symmetric planar division in epithelial cells^9^. More recently, studies performed in 3D mammary epithelial cell cultures indicate that EB1 is required for normal lumen formation^10^. Therefore, those findings position EB1 as an excellent candidate to act as an astral microtubule sensor for the cortical cues that determine spindle orientation in epithelial cells. How EB1 localization at spindle poles or astral microtubules is regulated has not been elucidated.

AKAP350 (AKAP450/CG-NAP) is a PKA anchoring protein that has a prominent role in the regulation of microtubule dynamics^11–13^. By recruiting components of the γ-tubulin ring complex (γ-TURC), AKAP350 participates in microtubule nucleation at the centrosome^11^, and at the Golgi apparatus^13^. In addition, AKAP350 regulates the kinetics of microtubule growth^12,14^; the mechanism involved, though, has not been clarified yet. We have previously shown that AKAP350 participates in the development of apical canalicular structures in hepatic epithelial cells and that Golgi derived microtubules were involved in this function^15^. In the present study we analyzed AKAP350 participation in the establishment and maintenance of epithelial polarity using 3D MDCK cell cultures. Our results demonstrate that AKAP350 ensures the formation of epithelial structures with a single lumen by participating in the regulation of spindle positioning. Furthermore, we provide evidence supporting that the mechanism defining spindle orientation involves AKAP350 dependent recruitment of EB1 to spindle poles and astral microtubules, thus giving insight into the spindle events that condition this process.

## Results

### Decrease of AKAP350 expression leads to spindle missorientation and defective cystogenesis

**Figure 1:**
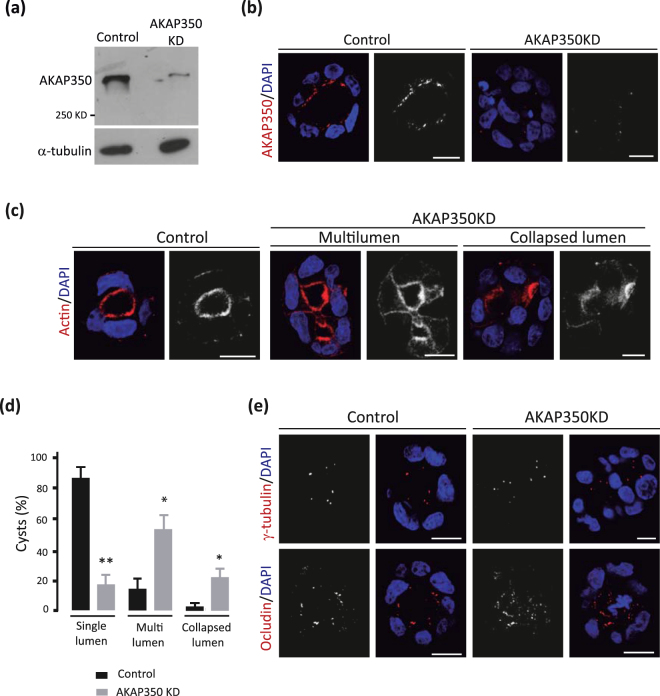
Decrease of AKAP350 expression induces defective lumen formation. AKAP350KD MDCK cells and control cells expressing non-specific small hairpin (sh)RNAs were generated as described in Materials and Methods. (**a**) Immunoblots showing AKAP350 expression in control and AKAP350KD cells and the corresponding loading control. (**b**–**e**) Control and AKAP350KD cells were grown in Matrigel for 72 h, stained and analyzed by confocal microscopy. Single confocal sections at the center of the cysts are shown. Merged images show AKAP350 (**b**), actin (**c**) and ocludin or γ-tubulin (**e**) staining in red and DAPI staining in blue in control and AKAP350KD cysts. (**d**) Bars represent the percentage of cysts that developed each lumen phenotype for at least 10 cysts analyzed in 6 independent experiments. Scale bars, 10 μm. *p < 0.05; **p < 0.01.

To assess AKAP350 role in the development of apico-basal polarity in columnar epithelial cells, we generated MDCK cells with decreased AKAP350 expression (AKAP350KD) using a lentiviral based short hairpin RNA expression system (Fig. 1a and Supplementary Figure 1a), as we have previously described^16^. Cells were grown on filters for 48 h, in order to induce polarization. In these conditions, 24 h after seeding, cells already develop tight junctions and polarized distribution of apico-basal markers^17^. Our results showed that the decrease in AKAP350 expression did not affect the polarized distribution of the tight junction protein occludin (Supplementary Figure 1b, top row). Similarly, the centrosome marker γ-tubulin displayed subapical localization regardless of AKAP350 levels (Supplementary Figure 1b, second row). In addition, AKAP350KD cells did not show any evidence of altered actin distribution (Supplementary Figure 1b, bottom row). Therefore, these results indicate that centrosome, tight junction and actin organization in 2D MDCK cell cultures were not affected by the decrease in AKAP350 expression.

In order to evaluate cystogenesis, AKAP350KD cells were seeded on Matrigel at low density and, 72 h later, stained and analyzed by confocal microscopy (Fig. 1b-e). We first analyzed AKAP350 distribution in cyst cells. In control cells, AKAP350 staining was concentrated below the luminal membrane (Fig. 1b), which is in accordance to AKAP350 localization at the centrosome and Golgi apparatus. As expected, AKAP350 staining was significantly decreased in AKAP350KD cysts. These staining also revealed that AKAP350 cysts presented morphological alterations. Cyst phenotype characterization using actin staining indicates that 85% of control cells developed normal cysts with a single lumen. In contrast, less than 20% of AKAP350KD cysts presented normal morphology, whereas the main fraction of AKAP350KD cells formed cysts with lumen anomalies (Fig. 1c,d). Lumen morphology abnormalities could be consequence of cell polarity defects and the resulting distortion of the apical membrane^4,18^. The analysis of γ-tubulin (Fig. 1e, first row) and occluding (Fig. 1e, second row) staining revealed that, even though lumen morphology was affected, the subapical centrosome localization and the apical tight junction organization were conserved in AKAP350KD cysts.

**Figure 2:**
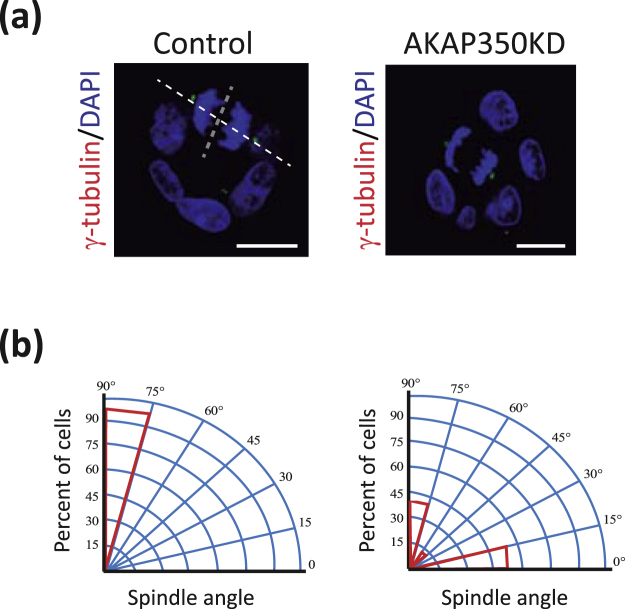
Reduction of AKAP350 expression leads to spindle missorientation. Cells were grown in Matrigel for 48 h and stained as indicated. Images were obtained by confocal microscopy, and spindle orientation in mitotic cells in cysts with a noticeable lumen analyzed as described in Materials and Methods. (**a**) Images show the projection of the central sections for γ-tubulin and DAPI staining corresponding to control and AKAP350KD cysts, where the spindle angle of anaphase cells has been delineated. Scale bars, 10 μm. (**b**) Circular section histogram graph. The poblational distribution of anaphase or telophase spindle angles in control and AKAP350KD cysts is indicated in red. Results are representative of three independent experiments.

Considering that formation of cysts with lumen abnormalities is frequently associated to spindle missorientation, we analyzed spindle alignment in AKAP350KD cysts. At the onset of mitosis, the angle formed by the spindle poles and the apicobasal axis is close to random, being the definitive spindle position achieved during metaphase^19,20^. Therefore, we considered cysts that had a noticeable lumen to analyze the spindle orientation in cells that were already in anaphase or telophase. The spindle poles were identified using γ-tubulin staining, and spindle orientation in mitotic cells measured as the angle formed by a line defined by the apico-basal axis, and the line that linked both spindle poles (Fig. 2a). Most dividing cells in control cysts oriented their spindles perpendicular to the apico-basal axis (82° ± 2°), whereas spindle orientation in AKAP350KD was flawed (32° ± 13°, p < 0.01) (Fig. 2b).Therefore, in AKAP350KD cells, defective lumen formation was associated to cell failure to orient their spindles symmetrically within the epithelial plane.

We analyzed if the alteration in spindle orientation observed in AKAP350KD cells could be secondary to defective microtubule nucleation at the spindle poles. Even though AKAP350 participates in microtubule nucleation in interphase centrosomes^11^, studies performed in HeLa and RPE1 cells showed that the decrease in AKAP350 expression induced by RNA interference did not elicit any defect in microtubule nucleation at the centrosomes^12,13^. We first analyzed microtubule nucleation in MDCK cells that had been subjected to nocodazole treatment. Our results showed that, after 15 min of nocodazole removal, the number of microtubules nucleated by the centrosomes in AKAP350KD cells was similar to that of control cells (Supplementary Figure 2a). We further analyzed if nucleation of astral microtubules was affected by the decrease in AKAP350 expression, by characterizing astral microtubules in control or AKAP350KD metaphase cells. We found that there was not a significant difference in the number (Supplementary Figure 2b), length or intensity (Supplementary Figure 2c) of astral microtubules in AKAP350KD cells. Therefore, these results indicate that the defective spindle orientation in AKAP350KD was not attributable to inefficient astral microtubule nucleation at the spindle poles.

### AKAP350 interacts with EB1 at the centrosomes in mitotic cells

**Figure 3:**
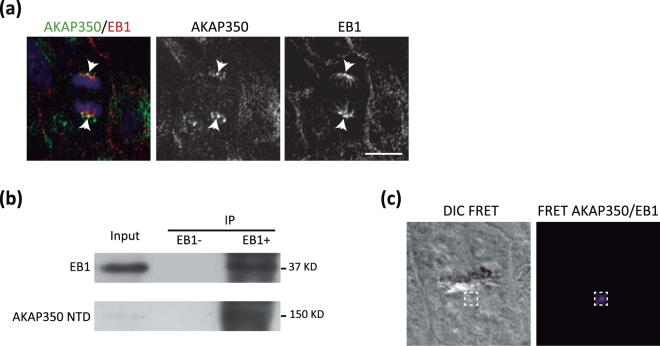
AKAP350 interacts with EB1 at the centrosomes. **(a**) Merged images show AKAP350 (green) and EB1 (red) staining in MDCK cells. Arrows indicate centrosomes in mitotic cells, where significant colocalization is observed. Scale bars, 10 μm. (**b**) MDCK cells with stable expression of AKAP350(1-1029) domain fused to GFP (AKAP350NTD) were prepared as described in Materials and Methods. AKAP350NTD cell lysates (Input) were incubated with an anti-EB1 antibody, and subjected to immunoprecipitation using protein A-sepharose beads. Non-bound material was removed, beads were washed and immunoprecipitates (IP) eluted with sample buffer. A negative control was run by incubating cell lysates with protein A-sepharose beads in the absence of anti-EB1 antibody (EB1-). Samples were analyzed by Western blot using anti-EB1 (top) and anti-GFP (bottom) antibodies. (**c**) Analysis of EB1/AKAP350 interaction *in situ* was performed using immuno-FRET by acceptor photobleaching, as described in Materials and Methods. The first image show the DIC channel of a mitotic cell, which was bleached at the centrosome area in the acceptor channel. The second image illustrates in fire pseudocolor the increase in intensity in the bleached area in the donor (AKAP350) channel, after photobleaching of the acceptor (EB1) channel.

In order to investigate how AKAP350 modulates spindle orientation, we looked for putative partners that could participate in this mechanism. Previous studies demonstrated that EB1 regulates spindle orientation in epithelial cells by mechanisms that are not completely clear [reviewed in^1^]. Mostly recognized as a microtubule plus end binding protein that shows a comet like distribution in interphase cells, EB1 also localizes to centrosomes, astral microtubules and kinetochore fibers in mitotic cells. We first analyzed if AKAP350 colocalized with EB1. We found that there was a clear EB1/AKAP350 colocalization at the spindle poles in mitotic cells (Fig. 3a). It has been reported that AKAP350 localizes to microtubule plus ends^14^. Nevertheless, we did not find any evidence of AKAP350/EB1 colocalization outside of the centrosome. AKAP350 interacts with p150glued^21^, the main subunit of the dynactin complex, which directly associates with EB1^22^, raising the possibility that they could be part of the same protein complex. We generated MDCK cells with stable expression of the AKAP350 domain responsible for AKAP350/p150 glued interaction, AKAP350(1-1029) (AKAP350NTD), expressed as a GFP fusion protein. To assess the interaction between AKAP350 and EB1, we performed coimmunoprecipitation experiments in AKAP350NTD cells (Fig. 3b). We found that AKAP350NTD was present in EB1 immunoprecipitates, thus indicating that, by means of its amino terminal domain, AKAP350 arranges a protein complex with EB1. We further analyzed the interaction between endogenous AKAP350/EB1 using immuno FRET by acceptor photobleaching. We performed those experiments using either EB1/AKAP350 or AKAP350/EB1 as donor/acceptor pairs. In both cases we found an increase in the donor fluorescence when the acceptor was bleached at the centrosome area (Fig. 3c), being the FRET efficiencies for EB1/AKAP350 pair 5% and for AKAP350/EB1 pair 11% (n = 6, p < 0.05). On the other hand, photobleaching of the acceptor channel at the centrosome area when the acceptor protein was not immunostained or when the pair EB1/γ-tubulin was used as control did not elicit any increase in the donor fluorescence intensity. These results give additional evidence of a direct interaction between AKAP350 and EB1 at the centrosomes.

### Reduction of AKAP350 levels results in decreased EB1 localization at spindle poles and astral microtubules

**Figure 4:**
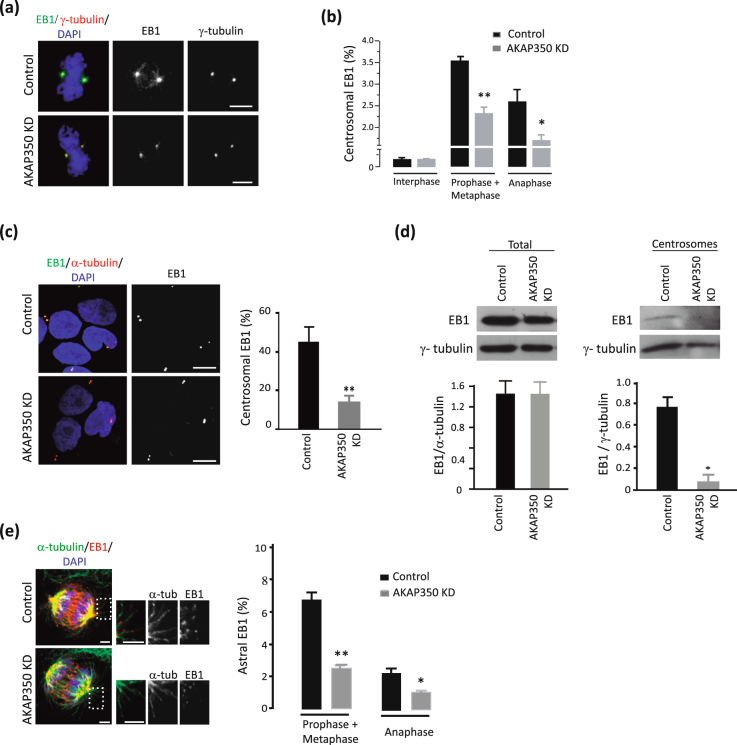
Reduction of AKAP350 levels results in decreased EB1 localization at spindle poles and astral microtubules. (**a**) Merged images show the visualization of EB1 (green) and γ-tubulin (red) staining in metaphase control and AKAP350KD cells. (**b**) Centrosomal localization of EB1in mitotic cells was determined by setting a threshold on the γ-tubulin channel to define a mask, which was used to automatically outline the centrosomal voxels, and related to total EB1 fluorescence, which was determined using a threshold on the EB1 channel to define a mask to outline total voxels for EB1 staining. Bars represent the percentage of EB1 fluorescence present at the spindle poles for interphase, prometaphase, and anaphase cells. Results are representative of at least 10 cells for each group analyzed in 3 independent experiments. (**c**) Cells were treated with nocodazole 10 μg/ml for 3 h, washed and dual stained with anti-α-tubulin and anti-EB1 antibodies. Merged images show the visualization of EB1 (green), α-tubulin (red) and DAPI (blue) staining in control and AKAP350KD cells. Bars denote the percentage of EB1 fluorescence present at the centrosome, representative of three independent experiments, calculated as in **b**. (**d**) Centrosomes enriched subcellular fractions were prepared by differential centrifugation of control or AKAP350KD cell lysates using a Fycoll gradient, as described in Materials and Methods. Total cells lysates and the centrosomal fraction were analyzed for EB1 expression by western blot. Bar graphs represent the density of the band corresponding to total or centrosomal EB1 relative to α-tubulin or γ-tubulin density, respectively, of three independent experiments. (**e**) Merged images show the visualization of EB1 (red), α-tubulin (green) and DAPI (blue) staining in control and AKAP350KD cells undergoing mitosis. The inset images show magnified views of astral microtubules (boxed areas). Bars represent the fraction of EB1 fluorescence present at astral microtubules. Scale bars, 5 μm (**a**,**c**) or 2.5 μm (**e**). *p < 0.05; **p < 0.001.

We further explored if AKAP350 was involved in EB1 recruitment to spindle poles, by examining EB1 levels at these organelles in AKAP350KD cells. We obtained immunofluorescence confocal microscopy images of control and AKAP350KD cells dually stained for EB1 and γ-tubulin (Fig. 4a), and measured the fraction of EB1 intensity of fluorescence that was present at the centrosomes. AKAP350KD interphase cells did not show any alteration in the fraction of EB1 that localized at the centrosome (Fig. 4b). We observed that, in control cells, EB1 localization at the centrosome markedly increased during prophase and metaphase (>20 times) and, even though it decreased during anaphase, remained widely over interphase levels (Fig. 4b). The increase in EB1 levels was markedly affected in AKAP350KD mitotic cells (Fig. 4a,b). Similarly to what happens at the onset of mitosis, interphase microtubules subjected to nocodazole treatment and washout suffer rapid remodeling. In order to reevaluate our observations regarding centrosomal localization in mitotic cells, we analyzed EB1 levels at the centrosomes in cells subjected to nocodazole treatment immediately after nocodazole washout. Analysis of EB1 and γ-tubulin dual staining showed that centrosomal levels of EB1 in AKAP350KD cells were decreased to 30% of control cells (Fig. 4c). We further analyzed by western blot EB1 levels in centrosome enriched subcellular fractions, derived from control or AKAP350KD cells subjected to nocodazole treatment (Fig. 4d). Estimates that considered the total protein recovery and EB1 band density for each fraction indicated that approximately 3% of total EB1was recovered at the centrosomal fraction. EB1 levels in total cell lysates were not affected by the decrease in AKAP350 expression (Fig. 4d, left). Remarkably, the EB1 yield in centrosome fractions in AKAP350KD was severely decreased, representing only the 0,2% of the total EB1 (Fig. 4d, right). It is noteworthy that AKAP350KD cells also exhibited decreased recovery of γ-tubulin in centrosome fractions, what is in accordance with AKAP350 participation in the organization of the γ-tubulin containing ring complex at this organelle^11^ and consistent with our immunofluorescence analysis (Fig. 4a). Nevertheless, even when related to γ-tubulin levels, EB1 recovery was significantly decreased in AKAP350KD centrosomal fraction (Fig. 4d, bars).

We further evaluated if the decrease in EB1 levels at the spindle poles was associated with changes in EB1 levels at astral microtubules (Fig. 4e). The presence of EB1 in astral microtubules was significantly decreased in mitotic AKAP350KD cells. Besides, there was a noticeable decrease in EB1 levels at kinetochore fibers. Altogether, our results indicate that AKAP350 participates in the recruitment of EB1 to the centrosomes in mitotic cells, and, concomitantly, conditions EB1 levels at spindle microtubules.

### EB1 overexpression rescues AKAP350KD induced defective spindle orientation

**Figure 5:**
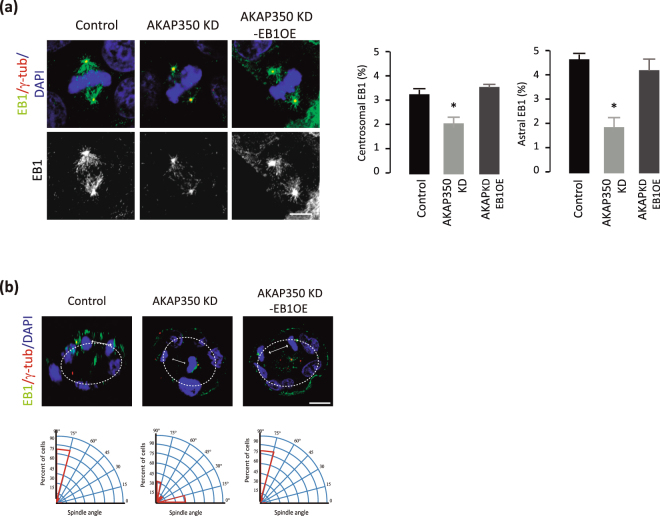
EB1 overexpression rescues AKAP350KD induced defective spindle orientation. MDCK cells with stable expression of EB1 fused to RFP (EB1OE) were generated and EB1OE with normal or decreased levels of AKAP350 (AKAP350KD-EB1OE) cells were prepared as described in Materials and Methods. (**a**) Merged images show the visualization of EB1 (green) and γ-tubulin (red) staining in metaphase control, AKAP350KD and AKAP350KD-EB1OE cells. Bars represent the percentage of EB1 fluorescence present at the centrosomes, defined at the γ-tubulin channel, and at astral microtubules in mitotic cells. Results are representative of at least 10 cells for each group analyzed in 3 independent experiments. Scale bar, 5 μm. (**b**) Cells were grown in Matrigel for 72 h and stained as indicated. Images were obtained by confocal microscopy, and spindle orientation in mitotic cells analyzed as described in Materials and Methods. Images show the projection of the central sections for γ-tubulin and DAPI staining corresponding to control, AKAP350KD cysts and AKAP350KD-EB1OE cysts, where the spindle angle of anaphase cells has been delineated, as explained for Fig. 2. Scale bar, 10 μm. The circular section histogram graphs show the population distribution of mitotic spindle angles in control, AKAP350KD and AKAP350KD-EB1OE cysts indicated in red. Results are representative of three independent experiments. *p < 0.05.

We evaluated if EB1 was actually implicated in the pathway leading to AKAP350KD induced alteration of spindle orientation, by analyzing the effect of increased expression of EB1 on spindle orientation in AKAP350KD cells. We generated control and AKAP350KD cells with stable overexpression of EB1, codified as a RFP fusion protein (EB1OE and AKAP350KD-EB1OE, respectively). We found that, in mitotic cells with decreased levels of AKAP350, EB1 overexpression restored EB1centrosomal and astral microtubule intensities to control levels (Fig. 5a). Previous studies reported that EB1 was overexpressed in various cancer types derived from epithelial cells, having been the association of EB1 with the malignant potential of tumor cells verified in hepatocellular carcinoma and in breast cancer cells^23–26^. In accordance with those studies, cystogenesis assays revealed that EB1 overexpression *per se* severely affected the formation of epithelial organotypic structures, leading to the formation of cysts with no lumen, cysts with altered polarity, and cysts that were more invasive (supplementary Figure 3). We used the population of AKAP350KD-EB1OE cysts which had a noticeable lumen to measure spindle orientation. As shown in Fig. 5b, we found that AKAP350KD-EB1OE recovered the spindle orientation perpendicular to the apico-basal axis (74° ± 7°), showing an average orientation in the range of spindle orientation in control cells (75° ± 6°) and significantly different from AKAP350KD cells (42° ± 10°, p < 0.05).

### AKAP350 delocalization from the centrosome induces decreased EB1 localization at spindle poles and astral microtubules, and defective cystogenesis

**Figure 6:**
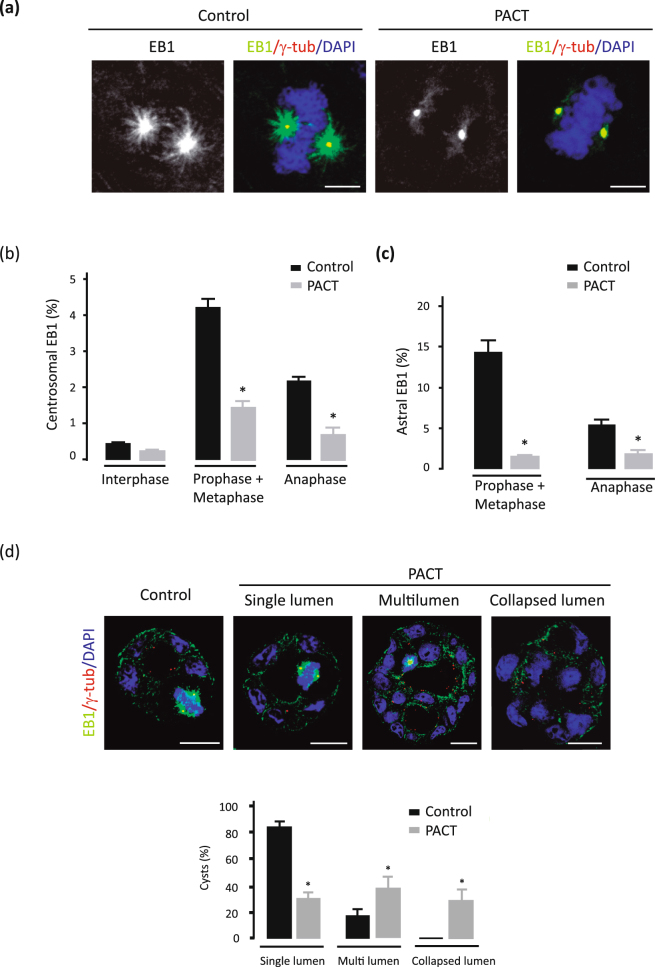
AKAP350 delocalization from the centrosome induces decreased EB1 localization at spindle poles and astral microtubules leading to defective lumen formation. MDCK cells with stable expression of the AKAP350(3386–3477) domain, which targets the protein to the centrosome, fused to GFP (PACT) and their respective controls stably expressing GFP were prepared as described in Materials and Methods. (**a**) Merged images show the visualization of EB1 (green), γ-tubulin (red) and DAPI (blue) staining in metaphase control and PACT cells. (**b**,**c**) Bars represent the fraction of EB1 fluorescence present at the spindle poles (**b**), or at astral microtubules (**c**) for interphase (**b**), prometaphase, and anaphase cells (**b**,**c**). Results are representative of at least 10 mitotic cells analyzed in 3 independent experiments. (**d**) Control and PACT cells were grown in Matrigel for 72 h, stained and analyzed by confocal microscopy. Single confocal sections at the center of the cysts are shown. Merged images show EB1 (green), γ-tubulin (red) and DAPI (blue) staining in control and PACT cells. Bars represent the percentage of cysts that developed each type of phenotype considering lumen morphology for at least 8 cysts analyzed in 4 independent experiments. Scale bars, 5 μm (**a**–**c**) or 10 μm (**d**). *p < 0.05.

In order to evaluate if the decrease in EB1 levels at astral microtubules observed in AKAP350KD cells was related to the reduction of its levels at the spindle poles, we prepared cells with stable expression of the carboxyl-terminal 92 aminoacid region of AKAP350 (AKAP350(3386–3477)), the pericentrin-AKAP450 centrosomal targeting (PACT) domain^27^. Expression of the PACT domain generates a reduction in AKAP350 localization at the centrosome without affecting total AKAP350 levels, or AKAP350 localization at the Golgi apparatus^15,16,27^. MDCK cells with stable expression of the PACT domain (PACT cells) were prepared by transducing MDCK cells with retroviral particles codifying for a PACT-GFP fusion protein (Fig. 6a). We first verified if AKAP350 presence at the centrosome was necessary for proper EB1 localization at this organelle. Similarly to what was observed in AKAP350KD cells, mitotic PACT cells showed a remarkable reduction in EB1 levels at their spindle poles (Fig. 6a,b). Therefore, we used this condition where EB1 was specifically delocalized from the spindle poles, to evaluate EB1 localization at astral microtubules. We found that PACT cells exhibited a significant decrease in EB1 localization at astral microtubules (Fig. 6c), thus supporting that the reduction of EB1 levels at the spindle poles may affect EB1 loading in nascent astral microtubules.

In order to analyze if delocalization of AKAP350/EB1 from the centrosome was sufficient for inducing defective lumen formation, we analyzed cystogenesis in PACT cells (Fig. 6d). PACT cells were cultured on matrigel at low confluence for 72 h. As expected, the majority of control cells formed normal cysts with a single lumen. On the other hand, only a minor fraction of PACT cells developed normal cysts, whereas most PACT cells formed cysts with more than one lumen, or with collapsed lumen. Accordingly, in contrast with the spindle orientation parallel to the epithelial sheet observed in most control cysts, many PACT cysts showed altered spindle orientation (Fig. 6d).

Therefore, centrosomal AKAP350 is involved in the recruitment of EB1 to the spindle poles, what has a direct impact on EB1 localization at astral microtubules. In accordance with these observations, the decrease in centrosomal AKAP350 levels leads to the development of cysts with phenotypes compatible with abnormal spindle orientation.

### Interference with AKAP350/EB1 interaction leads to defective cystogenesis

**Figure 7:**
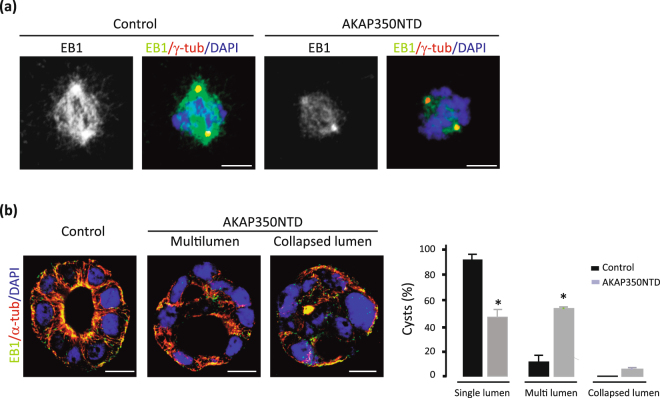
Interference with AKAP350/EB1 interaction is sufficient to induce defective cystogenesis. (**a**) Images show EB1 (green) and γ-tubulin (red) staining in control and AKAP350NTD mitotic cells. (**b**) Control and AKAP350NTD cells were grown in Matrigel for 72 h, stained and analyzed by confocal microscopy. Single confocal sections at the center of the cysts are shown. Merged images show EB1 (green), α-tubulin (red) and DAPI (blue) staining in control and AKAP350NTD cells. Bars represent the percentage of cysts that developed each type of phenotype for at least 8 cysts analyzed in 4 independent experiments. Scale bars, 10 μm. *p < 0.05.

To further confirm if EB1 interaction with AKAP350 at the centrosome was necessary for normal cystogenesis, we used cells with stable expression of the AKAP350NTD domain, which contains the AKA350/EB1 interaction region (Fig. 3), but not the PACT domain and, therefore, does not localize to the centrosome. We first evaluated if, effectively, AKA350NTD cells had decreased levels of EB1 at the centrosome. Cells were stained with anti-γ-tubulin and anti-EB1 antibodies (Fig. 7a), and EB1 levels at the centrosomes in mitotic cells analyzed as described above. EB1 localization at the centrosome was significantly decreased in AKAP350NTD cells, confirming that AKAP350NTD domain acts as a dominant negative protein regarding EB1 interaction with AKAP350 at the centrosome. The analysis of cystogenesis in AKAP350NTD cells revealed that, similarly to what was observed in AKAP350KD and PACT cells, AKAP350NTD cells developed an increased fraction of cysts with lumen abnormalities (Fig. 7b). Altogether, our results indicate that EB1 interaction with AKAP350 at the centrosome is necessary for normal cystogenesis.

## Discussion

The organization and maintenance of epithelial sheets require individual cell division to occur within the plane of the sheet. The relevance of accurate cell division orientation is underscored by studies reporting that the failure of epithelial cells to orient their spindle parallel to the epithelial plane is often associated to pathological conditions, such as intestinal tumors and polycystic kidney disease^28,29^. Key studies revealed the participation of cell-cell adhesion complexes in the specification of planar spindle orientation^8^. Many of the molecules involved in adherent junction interaction with the spindle astral microtubules have been identified, and the mechanism underlying their recruitment at this site has been uncovered^1,5^. Far less is known about the molecular actors that regulate spindle orientation at the spindle apparatus. Studies performed in Drosophila epithelium indicate that the fly homologue of EB1 is required for proper spindle alignment and symmetric epithelial division along the planar axis^9^. Even though spindle positioning was not measured, experiments in 3D primary cultures of mammary epithelial cells showed that EB1 depletion lead to formation of cysts with collapsed lumen^10^, what is compatible with altered orientation of cell division. Although the exact mechanism mediating EB1 regulation of spindle positioning has not been uncovered, the fact that EB1 is responsible for microtubule plus ends interaction with several proteins involved in astral microtubules stabilization and spindle orientation makes EB1 an excellent astral candidate for sensing cortical cues which can direct spindle orientation in epithelial cells^30^. For instance, EB1 mediates p150glued interaction with microtubule plus ends^22,31^, and, therefore could condition the dynein association with these structures, what constitute a crucial event in spindle positioning^5,9^. In this study, using organotypic 3D culture of MDCK cells we identified centrosomal AKAP350 as an important element in the specification of the axis of cell division. Furthermore, our results reveal that AKAP350 interacts with EB1 at the spindle poles, and indicate this interaction is critical for proper EB1 localization at the spindle apparatus.

Our previous studies demonstrated that loss of AKAP350 function leads to impaired apical actin cytoskeleton nucleation and decreased formation of apical “canalicular” structures in hepatocytes, and provided evidence that this effect is mediated by AKAP350 dependent microtubule nucleation at the Golgi apparatus^15^. Hepatocytes are epithelial hepatic cells that develop multiple apical poles, thus differing from most epithelial cells, including kidney tubule epithelial cells and enterocytes, which develop a single apical pole. In contrast with our findings in hepatocyte-epithelial cells, in the present study we found no evidence of defective development of the apical pole in 2D cultures of kidney tubule derived MDCK cells with decreased AKAP350 expression. Ls174T-W4 cells derive from the Ls174T colon epithelial cell line, which have been genetically engineered to produce an enterocyte-like phenotype in absence of cell-cell or cell-extracellular matrix contact^32^. Similarly to our studies in MDCK cells, our previous observations in Ls174T-W4 cells indicated that, even though the decrease in AKAP350 expression was associated to defective microtubule nucleation at the Golgi apparatus, it did not interfere with the development of the actin rich apical structures (brush borders)^33^. Therefore, our present studies provide additional evidence supporting that, compared with cells with hepatocyte type of epithelial polarity, epithelial cells with simple polarity have different requirements regarding the protein complexes that regulate their cytoskeleton dynamics during the initial events that condition the development of their apico-basal asymmetry.

During the development of different types of cell polarity, the non-central positioning of the centrosome primes very dissimilar effects. Nevertheless, much of the mechanics of movement and the regulatory mechanisms governing the asymmetric localization of the centrosome are highly similar [reviewed in^34^]. Previous studies demonstrate that, during the development of the immunological synapse, AKAP350 is involved in centrosome movement towards the site of T cell interaction with its target cell^35^. In addition, our previous studies indicated that AKAP350 participates in the polarized localization of the centrosome towards the front pole in migratory cells^16^. In contrast, our present study demonstrates that AKAP350 expression is not necessary for proper centrosome localization at the apical pole of MDCK, which is the first stage for the formation of the apical cilia. These results are also in agreement with our findings in Ls174T-W4 cells^33^, thus unveiling significant differences in the machinery involved in centrosome positioning during the development of epithelial polarity, when compared with immune synapse and migratory polarity.

In spite of not having effect on cell polarity in 2D cultures, 3D MDCK cell cultures revealed an essential role of AKAP350 on the specification of the location of the apical pole, which was associated to its effect on ensuring correct spindle orientation. Similarly, cdc42 has a central role in determining lumen formation by regulating mitotic spindle alignment, but does not lead to a conspicuous phenotype in 2D MDCK cell cultures^4,36^. In our model, the analysis of spindle positioning in 2D MDCK cultures revealed no evidence of alterations of spindle orientation with respect to the cell-substrate adhesion plane (data not shown). Therefore, these results prove that, at least in some cases, the 2D epithelial cell models have inherent limitations to reveal defects in cell division orientation.

Pericentrin is a centrosomal protein which is evolutionary and functionally related to AKAP350^37^. It has been demonstrated that pericentrin organizes a centrosomal complex which regulates spindle orientation in fibroblasts^38^. That study revealed that 2D cultures of pericentrin knocked out cells showed spindle misorientation relative to the cell-substrate adhesion plane, which was associated to a dramatic reduction in astral microtubules. Astral microtubules, and overall microtubule nucleation, were preserved in AKAP350KD cells (Supplementary Figure 2) and, as stated above, spindle orientation relative to the cell-substrate plane was not affected. Therefore, the mechanisms underlying our observations are clearly different from that involved in spindle mispositioning in pericentrin knocked out cells.

Regarding the mechanism involved in AKAP350KD induced defective cystogenesis, we found that AKAP350 recruits EB1 to the spindle poles, and that the decrease in AKAP350 expression leads to decreased EB1 levels not only at the spindle poles, but also at astral microtubules. As discussed above, several studies position EB1 as an excellent candidate to act as an astral microtubule sensor of cortical signals that could ensure accurate alignment of cell division in epithelial cells. Nonetheless, Gierke and Wittmannin showed that EB1 depletion only elicited a subtle increase in the number of cysts with collapsed lumen in MDCK cells^39^. As the authors discuss, the absence of a more prominent phenotype in EB1 knocked down cells could be due to a partial compensation by another member of this family, EB3, which can show redundancy with EB1^40^. In order to evaluate if EB1 was actually acting in the pathway by which AKAP350 ensured spindle orientation, we analyzed the effect of EB1 overexpression on spindle orientation during cystogenesis in AKAP350KD cells. We found that EB1 overexpression rescued the defective spindle orientation induced by AKAP350 depletion, thus confirming that EB1 participates in spindle orientation in MDCK cells, and that it was acting in the same pathway that AKAP350. According to the results illustrated in Figs 3 and 4, it is possible that EB1 would have not been able to restitute spindle orientation if AKAP350 was completely absent from the centrosome. Nevertheless, the presence of low levels of AKAP350 remaining at the centrosomes in AKAP350KD cells (Supplementary Figure 1) could have been enough to ensure EB1 scaffold at the centrosomes when EB1 levels were enhanced by the overexpression of the fusion protein.

The mechanism mediating AKAP350 dependent EB1 recruitment to astral microtubules is not clear. It was reported that AKAP350 could localize to microtubule plus ends^14^. Even though it has been definitely demonstrated that EB1 binds autonomously to microtubule plus ends^41^, it could be speculated that AKAP350 might be involved in the regulation of its stability by directly interacting with EB1 at this location. Nevertheless, we could not get evidence of AKAP350/EB1 colocalization at microtubule plus ends in our system. Furthermore, our results also demonstrated that the specific delocalization of the AKAP350/EB1 complex from the centrosome lead to decreased EB1 levels at astral microtubules. Hence, these results indicate that EB1 localization at astral microtubules is dependent on its recruitment to the spindle poles by AKAP350. Previous studies indicated the presence of EB1 comets associated to spindle poles, which tracked away from these organelles, suggesting the existence of EB1 association to microtubule plus ends at the centrosome^42^. Therefore, these results are compatible with a scenario where astral microtubule associated EB1 could be initially originated at the spindle poles. In this model, AKAP350 would be responsible for ensuring proper loading of EB1 to nascent mitotic microtubules plus ends. This hypothesis is in agreement with previous studies which showed that growth of newly formed microtubules was delayed in AKAP350KD cells^12^, and that formation of EB1 comets was reduced in cells with decreased AKAP350 expression^14^. Further studies will be necessary to confirm or to refute this model.

Decreased localization of EB1 at mitotic spindles could be sufficient to explain the deficiency in cystogenesis induced by defective AKAP350 expression. Nevertheless, AKAP350 could be involved not only in the recruitment of EB1 to the spindles, but also in the regulation of its phosphorylation state. Phosphorylation of plus end binding proteins has been proposed to be a mechanism for modulating the astral interaction with cell cortex molecules that precedes astral microtubule stabilization and pulling [reviewed in^26^]. In fact, phosphorylation of EB1 by the mitotic kinase ASK1 at the spindle poles was proposed to be essential for regulating EB1 affinity for astral microtubules and, concomitantly, spindle orientation in 2D cell cultures^43^. Furthermore, at the centrosome, AKAP350 also interacts with several kinases and phosphatases, including the casein kinase δ. EB1 is a substrate for this kinase, and their functional interaction is relevant for the centrosome positioning towards the immune synapse in T cells^44^. Therefore, our results are compatible with a scenario where AKAP350 recruits EB1 to the spindle poles positioning this protein close to specific kinases and/or phosphatases which could condition its affinity for specific partners at spindle microtubules or at the cell cortex.

In conclusion, our study demonstrates that centrosomal AKAP350 is essential for ensuring a precise spindle position during the formation of epithelial organotypic structures and provides evidence of the interaction between AKAP350 and EB1 at the spindle poles. Furthermore, our results reveal the functional relationship between AKAP350 localization at the spindle poles, and EB1 association to astral microtubules, thus uncovering interesting mechanistic data on the factors that govern spindle orientation during epithelial organogenesis.

## Materials and Methods

### Cell culture

MDCK cells were obtained from Keith Mostov lab (UCSF, CA).Cells were grown on plastic dishes in Dulbecco’s modified Eagle’s medium (DMEM) with 4.5 g of glucose/l, supplemented with 10% fetal bovine serum and antibiotics. Cells were tested monthly by PCR for contamination with mycoplasma.

### Reduction of AKAP350 expression

In order to reduce AKAP350 expression in MDCK cells, we proceeded as we have described previously^16^. Constructs were made by annealing and ligating oligonucleotides targeting a specific AKAP350 sequence (5′-GCAAGAACTAGAACGAGAA-3′) or a scrambled control into the AgeI and EcoRI cloning sites of pLKO.1-puro vector (details at http://www.addgene.org). These constructs were sequenced and used to co-transfect human embryonic kidney 293 FT cells with Virapowerlentiviral packaging mix (Invitrogen, Carlsbad, CA). The next day, transfection complexes were removed, and cells were allowed to produce virus for 24 h. Media containing virus were collected and used to directly transduce MDCK cells overnight. The cells were allowed to recover for 24 h and subjected to puromycin selection (5 μg/ml) for 2 weeks. Silencing was confirmed by western blotting and immunofluorescence analyses.

### Generation of stable cell lines

The AKAP350(1-1029) domain fused to GFP was donated by James R. Goldenring (Cell and Developmental Biology,Vanderbilt University). The plasmid codifying for EB1 fused to RFP was kindly provided by Alfredo Cáceres (Instituto de Investigaciones Médicas Mercedes y Martín Ferreyra-CONICET). MDCK cells were transfected by using lipofectamine 3000 (Invitrogen). After 24 h, the antibiotic Geneticin (Invitrogen, 500 μg/ml) was added to the medium to select the transfected cells. These cell lines were maintained in a medium containing Geneticin (200 μg/ml) for 2 weeks in conditions that were otherwise similar to those used to maintain parental cells. Individual clones of cells stably expressing AKAP350NTD–GFP (AKAP350NTD) or GFP (control), and EB1-RFP or RFP (control) were prepared by using serial dilutions of the selected cells.

The AKAP350CTD domain cloned into pEGFP-C1^14^ was used as a template to clone theAKAP350 3386–3477 (equivalent to AKAP450 3699-3790) domain (PACT) into the retroviral vector pLPC (Clontech). The following primers were designed containing linkers for the restriction enzymes EcoRI and XhoI: FW: 5′-AAAGAATTCTTAAAGAGAATTTATGGTAAATAC-3′ and RV: 5′-AAACTCGAGTTATGTGACTCGATGCCACCGT-3′. GP2-293 cells cultured in DMEM 10% FBS were transfected using the calcium phosphate method with a particle suspension containing 4 μg of pENV and 4 μg of pLPC-GFP or pLPC-GFP PACT in125 mM CaCl2/HBS (25 mMHepes pH 7, 140 mM NaCl, 0.75 mM Na_2_HPO_4_). After 8 hours of incubation the medium was changed and the cells were further incubated for further 48 hours. Retrovirus supernatants from the GP2-293 plates were collected, 500 μl of FBS and 8 μg/ml polybrene were added and the cell debris and aggregated virus were removed by filtering the supernatant through a 0.45 µm cellulose acetate filters. Filtered Polybrene-containing pLPC-PACT or pLPC retrovirus supernatants were added to MDCK cells. After 24 h, the media was changed and the following day stable cells were selected by adding 5 μg/ml of Puromycin.

### Inmunofluorescence

Cells were grown on glass coverslips, washed with PBS and fixed with 4% paraformaldehyde at room temperature or in 100% methanol at −20 °C. Fixed cells were permeabilized and blocked with 0.3%Triton X-100/1% bovine serum albuminin/PBS, pH7.4, for 10 min. Then, they were incubated with rabbit monoclonal antibodies against EB1 (Abcam-ab50188, 1:400), occluding (Zymed, 1:60), and mouse monoclonal antibodies against AKAP350 (1:80)^45^, γ-tubulin (Sigma-T6557, 1:250), α-tubulin (Sigma-T9026, 1:300), for 2 h. The coverslips were washed, incubated for 1 h with the secondary fluorescence-conjugated antibodies conjugated to Alexa 488, Alexa 560 or Alexa 633 or phalloydin-Alexa 568 (Molecular probes-A34055, 1:200) for actin staining and with 4′,6-diamidino-2-phenylindole (DAPI) for nuclear staining and mounted with ProLong. Fluorescence was detected by using confocal laser microscopy (Nikon C1SiR with inverted microscope Nikon TE200). Serial optical 0.3-μm (for 2D cultures) or 0.5 μm (for cyst assays) thick sections were collected in the z-axis. z-stacks were built, and projections were obtained using ImageJ tools. In preparing the figures, adjustment in brightness and contrast were equally applied to the entire images using Adobe Photoshop software to improve visualization of fluorescence.

### Imaging FRET measurements

In the present study, the acceptor bleaching FRET method was used, as described in detail^46^, using a LSM880 confocal with an ObserverZ1 inverted microscope. Two FRET donor/acceptor combinations were probed: anti-EB1 antibody recognized with secondary antibody labeled with Alexa 555 as a donor or with Alexa 633 as an acceptor, and anti-AKAP350 antibody recognized with secondary antibody labeled with Alexa 555 as a donor or with Cy5 as an acceptor. Controls were performed labeling exclusively the donor channel, in the absence of the acceptor fluorophore. As negative control, we labeled the samples with an anti-EB1 antibody recognized with an Alexa 555 conjugated antibody, and anti-γ-tubulin recognized with a Cy5 labeled secondary antibody, in order to get a donor/acceptor pair that colocalized at the centrosome but did not interact. The energy transfer was detected as an increase in donor (Alexa 555) fluorescence (dequenching) after photobleaching of the acceptor (Cy5 or Alexa 633) fluorophore. The validity of using the acceptor photobleaching technique to quantify FRET is based on the fact that the only factor that can lead to a difference in donor fluorescence in the presence and absence of acceptor is energy transfer. The FRET experiments were performed using secondary antibodies at a 1 donor: 2 acceptor ratio. Both acceptor dyes were photobleached using the 635-685 band of a He-Ne laser (100% power, 70 iterations of 130 ms). Under these conditions, ∼97% of the Cy5 or ∼30% of Alexa 633 were bleached from the entire centrosomal region. Three images of the Alexa 555 (donor) channel were acquired pre bleaching and three images of the same field were taken after photobleaching. No increase in the donor channel fluorescence intensity occurred in control samples labeled only with Alexa 555 or in the negative control. FRET efficiency was calculated as: (IDa-IDb)/IDa, being IDa and IDb the fluorescence intensity of the donor channel at the quenched area after and before the photobleaching, respectively.

### 2D and 3D MDCK culture

For analysis of epithelial polarity in 2D MDCK culture, cells were grown on transwells containing 0.4 um pore size polycarbonate membrane inserts (Corning Inc.) for 2 days. MDCK cells were fixed to analyze cell polarization by immunofluorescence. Cells were stained and analyzed by confocal microscopy as described above.

For cystogenesis analysis, MDCK cells were seeded as single-cell suspension (8.10^4^ cells per ml) in Matrigel (BD Biosciences). After 2 or 3 days, the gels were washed with PBS and fixed (4% paraformaldehyde, 30 min at room temperature or methanol, 7 min, −20°C). Cells were pre-treated with 0.5% Triton X-100/PBS (10 min, 4 °C) and then permeabilized and blocked with 1% serum albumin/0.2%Triton X100/0.05% Tween/PBS30 min at room temperature. Incubations with antibodies were performed in blocking solution for a minimum of 2 h at RT. Images were obtained by confocal microscopy as described above.

### Inmunoprecipitation

AKAP350NTD cells grown on plastic were washed, scrapped and lysed by the addition of lysis buffer (1% Triton X-100, 10% glycerol, 137 mM NaCl, 2 mM EDTA, 20 mMTris-HCl), supplemented with protease inhibitors. Cell lysates (300 μg/sample) were incubated overnight with 5 μl of anti-EB1 or without antibody (control) under constant agitation at 4 °C. Protein A-Sepharose (Sigma Chemical Co.) was added to each tube to reach a concentration of 4 mg/ml in a final volume of 200 μl and samples were further incubated for 2 h. After that, samples were centrifuged 5 min at 5,000 rpm. Pellets were washed three times with PBS and proteins bound to the Protein A-Sepharose were dissolved in 20 μl sample buffer and heated at 90 °C for 10 min. The complete volumes of these recovered immunoprecipitates and 10% of their supernatants (non-bound material) were loaded and subjected to immunoblot analysis.

### Immunoblotting

Cells were washed with cold PBS, scraped and pelleted at 200 *g* for 5 min at 4 °C. Pelleted cells were resuspended in Triton X-100 1% in PBS pH 7.4 with protease inhibitors, and subjected to two freeze–thaw cycles. Lysates were centrifuged at 1000 *g* for 5 min, and the clear supernatants were conserved. Total protein concentrations were measured according to Lowry *et al*. (1951). Samples were heated for 10 min at 70 °C in sample buffer (20 mMTris-HCl, pH 8.5, 1% SDS, 400 µM DTT, 10% glycerol). Then, they were subjected to SDS polyacrylamide gel electrophoresis (4–10% gradient to verify AKAP350 silencing or 10% otherwise). The proteins in the 4% gel were transferred to nitrocellulose membranes (Amersham Pharmacia Biotech), whereas the 10% gel proteins were transferred to polyvinyl difluoride membranes (Perkin Elmer Life Sciences). Blots were blocked with 5% nonfat dry milk in PBS, 0.3% Tween-20 (PBS-Tween). Nitrocellulose blots were then probed with the primary antibody mouse anti-AKAP350 (1:500), and polyvinyl difluoride membranes were probed with mouse monoclonal antibodies against α-tubulin (1:5000), and rabbit monoclonal antibodies against EB1 (1:1000) or GFP (1:500, Santa Cruz). The blots were then washed and incubated with the horseradish peroxidase-conjugated corresponding secondary antibodies, and bands were detected by enhanced chemiluminescence (Pierce, Thermo Scientific). Autoradiographs were obtained by exposing the blots to Kodak XAR film. The bands were quantitated by densitometry using the NIH Image J program.

### Analysis of EB1 localization

EB1 localization in mitotic cells. Images of EB1 and γ-tubulin or α-tubulin co-staining were obtained by confocal microscopy, as described above, and analyzed using appropriate ImageJ plugins. Mitotic cells undergoing pro-metaphase and anaphase were identified according to the level of chromosome segregation. After removing the background, centrosomal localization of EB1in those cells was determined by setting a threshold on the γ-tubulin channel to define a mask, which was used to automatically outline the centrosomal voxels. Localization of EB1 at astral microtubules was determined by defining a mask in the EB1 channel which included the voxels corresponding to the entire spindle apparatus, and clearing out from the region of interest defined by that mask the voxels corresponding to the centrosomes and the kinetochore fibers, which were defined in γ−tubulin or α-tubulin channels. Intensity of EB1 fluorescence at the centrosomes or at spindle asters were related to total EB1 fluorescence, which was determined using a threshold on the EB1 channel to define a mask to outline total voxels for EB1 staining.

EB1 localization at the centrosome in nocodazole treated cells. In order to analyze centrosomal EB1 in an additional condition associated to conspicuous microtubule remodeling, cells were subjected to chemical disruption of microtubules induced by treatment with 33 μM Nocodazol for 2 h in ice and 1 h at 37 °C. After nocodazole treatment, cells were washed with PBS, fixed, stained and analyzed as described for mitotic cells. Alternatively, centrosome-enriched fractions were prepared by centrifugation on a Fycoll cushion, using a method based on Blomberg-Wirschell and Doxsey^47^. Briefly, cells were treated with Nocodazole (1.65 μM) and Cytochalasin (390 ηΜ) for 30 min, and washed with PBS. Cells were resuspended and gently sonicated in lysis buffer containing 1mMTRIS-HCl [pH 8], 0.5% β-Mercaptoethanol, 0.5% Triton X-100 and protease inhibitors. Components were added to cell lysates to achieve a final concentration of 10 mM PIPES, 1 mM EDTA. In order to eliminate unbroken cells and nuclear fractions, cell extracts were centrifuged at 1,500 *g* for 15 min. The supernatant was filtered through a nylon mesh and loaded on the top of a 20% (w/v) Ficoll cushion in buffer 10 mM PIPES/1 mM EDTA with 0.1% Triton X-100 and tubes were spun at 25,000 g in a swinging bucket rotor. Centrosomes were taken from the top of the Ficoll cushion using a Pasteur pipet, and EB1 levels at this fraction analysed by western blot.

### Analyzis of microtubule nucleation

MDCK cells were seeded 24 h before the experiment, incubated with 8 μM Nocodazol for 2 h in ice and 1 h at 37 °C and allowed to recover at 37 °C for 15 min. For analysis of centrosome nucleation of microtubules, cells were immediately treated 45 sec with extraction buffer (60 mM PIPES, 25 mM HEPES, 10 mM EGTA, 2 mM MgCl2, 0.1% Saponin, pH 6.9, supplement with 0.25 nM nocodazole and 0.25 nM paclitaxel). Cells were then fixed and stained as described in Immunofluorescence Confocal Microscopy. Microtubule nucleation was analysed using the Microtubule tools plugin of the ImageJ software.

### Statistical analysis

Data are expressed as mean ± s.e.m. and are representative of at least three experiments. Paired Student’s t-test was used for comparison between experiments or for comparisons within each experiment when mixed populations of cells were used. Otherwise, non-parametric Mann–Whitney test was used for comparisons within each experiment. P < 0.05 was considered statistically significant.

## Electronic supplementary material

Supplementary material
