# Supplementary material for: Akap350 Recruits Eb1 to The Spindle Poles, Ensuring Proper Spindle Orientation and Lumen Formation in 3d Epithelial Cell Cultures
Source: Sci Rep. 2017 Nov 2;7:14894. doi: 10.1038/s41598-017-14241-y (PMC5668257; doi:10.1038/s41598-017-14241-y)
Supplement: Supplementary file 1 — Supplementary material [file 41598_2017_14241_MOESM1_ESM.pdf]

AKAP350 RECRUITS EB1 TO THE SPINDLE POLES, ENSURING PROPER SPINDLE ORIENTATION AND LUMEN FORMATION IN 3D EPITHELIAL CELL CULTURES. Evangelina Almada, Facundo M. Tonucci, Florencia Hidalgo, Anabela Ferretti, Solange Ibarra, Alejandro Pariani, Rodrigo Vena, Cristián Favre, Javier Girardini, Arlinet Kierbel, and M. Cecilia Larocca.- SUPPLEMENTARY INFORMATION

Supplementary Figure 1. Reduction of AKAP350 levels does not affect epithelial polarity in 2D MDCK cultures. AKAP350KD MDCK cells and control cells expressing non-specific small hairpin (sh)RNAs were generated as described in Materials and Methods. (**a**) Images obtained by immunofluorescence confocal microscopy showing AKAP350 expression and distribution in control and AKAP350KD cells. (**b**) Control and AKAP350KD cells were seeded at confluence on filters and, after 48 h of cell culture, fixed and stained. The polarized distribution of occludin (first row), γ-tubulin (second row) and actin (third row) was analyzed by confocal microscopy. Images are z-sections representative of at least 4 fields analyzed in three independent experiments. Scale bars, 10 μm.

Supplementary Figure 2. Reduction of AKAP350 levels does not affect microtubule nucleation at the spindle poles. (**a**) Control and AKAP350KD MDCK cells were subjected to nocodazole recovery treatment as described in the Materials and methods section. Images show the output of the ImageJ plugin “Microtubule tools”, which allow the identification of individual microtubules. Bars represent the average number of individual microtubules nucleated by 20 cells in each group. (**b**) Merged images show the visualization of EB1/α-tubulin staining in metaphase control and AKAP350KD cells. The outputs of aster microtubule analysis performed with the ImageJ plugin are also shown as in A. Bars represent the average number of astral microtubules for 10 cells in each group. (**c**) Images show α-tubulin staining in metaphase control and AKAP350KD cells. Bars represent average microtubule length and media α-tubulin intensity of fluorescence associated to astral microtubules, for at least 10 cells. Scale bars, 5 μm.

Supplementary Figure 3. EB1 overexpression promotes abnormal cystogenesis. Cells with stable overexpression of EB1 were grown in Matrigel for 72 h and stained as indicated. Images obtained by confocal microscopy show cysts with no lumen (first row) and with invasive phenotypes (second and third row). Scale bars, 10 μm.

Supplementary Western blot images. Images show the unedited scans of the western blots corresponding to Figure 3 (**a**) and Figure 4 (**b**). (A) AKAP350NTD cell lysates (Input) were incubated with an anti-EB1 antibody, and subjected to immunoprecipitation using protein A-sepharose beads. Non-bound material (NB) was removed, beads were washed and immunoprecipitates (IP) eluted with sample buffer. The control of immunoprecipitation specificity was prepared by performing the first incubation in the absence of the anti-EB1 antibody. Samples were analyzed by Western blot using anti-EB1 (top) and anti-GFP (bottom) antibodies. Arrows indicate the immunoreactive band that best fit the predicted molecular weight for EB1 (30 kDa) and AKAP350NTD-GFP fusion protein (157 kDa). (**b**) Centrosomes enriched subcellular fractions were prepared by differential centrifugation of control or AKAP350KD cell lysates using a Fycoll gradient, as described in Materials and Methods. Cell lysates and centrosomal enriched fractions were analyzed for EB1 and γ-tubulin expression by western blot.

Supplementary Figure 1

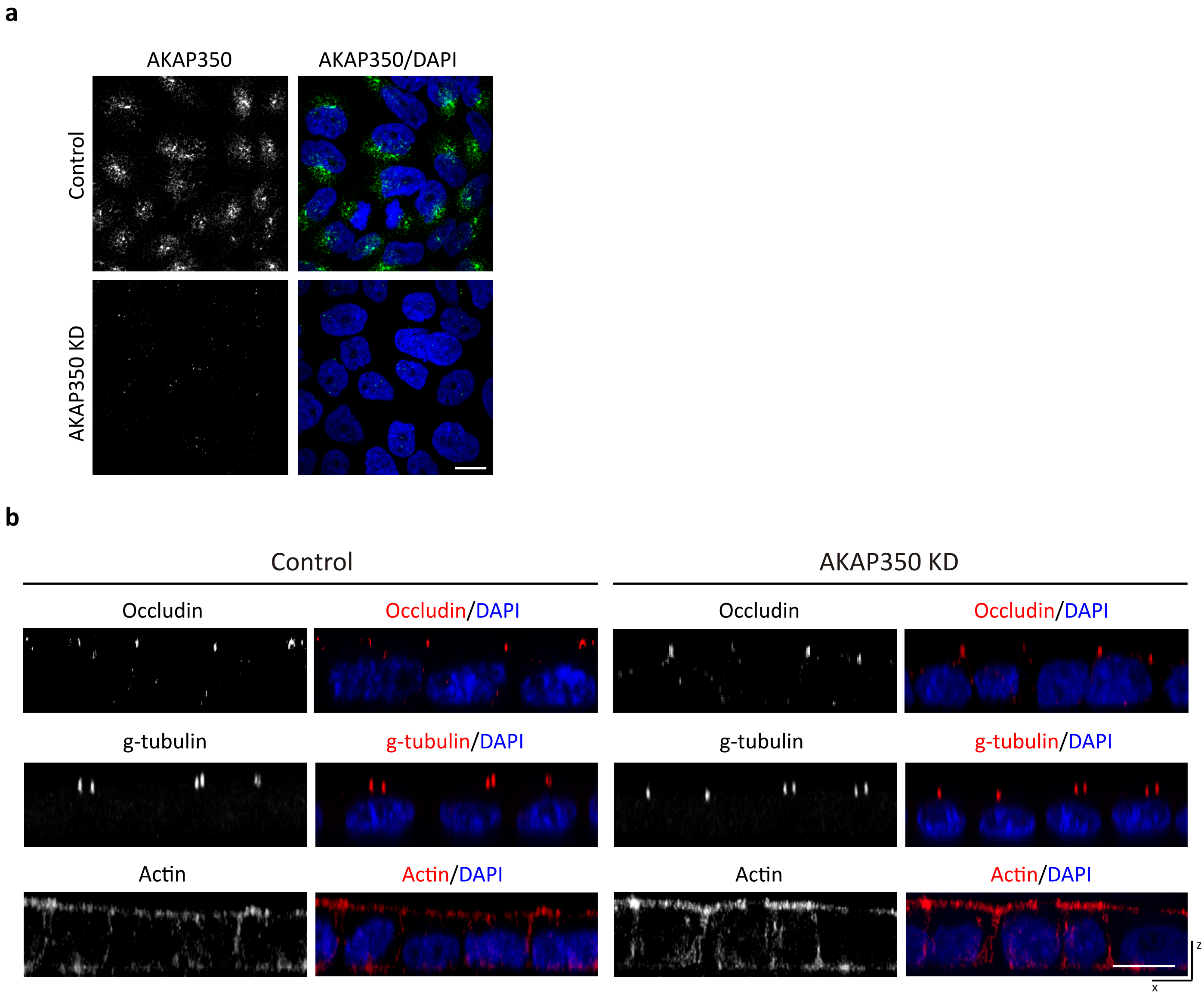

Supplementary Figure 2

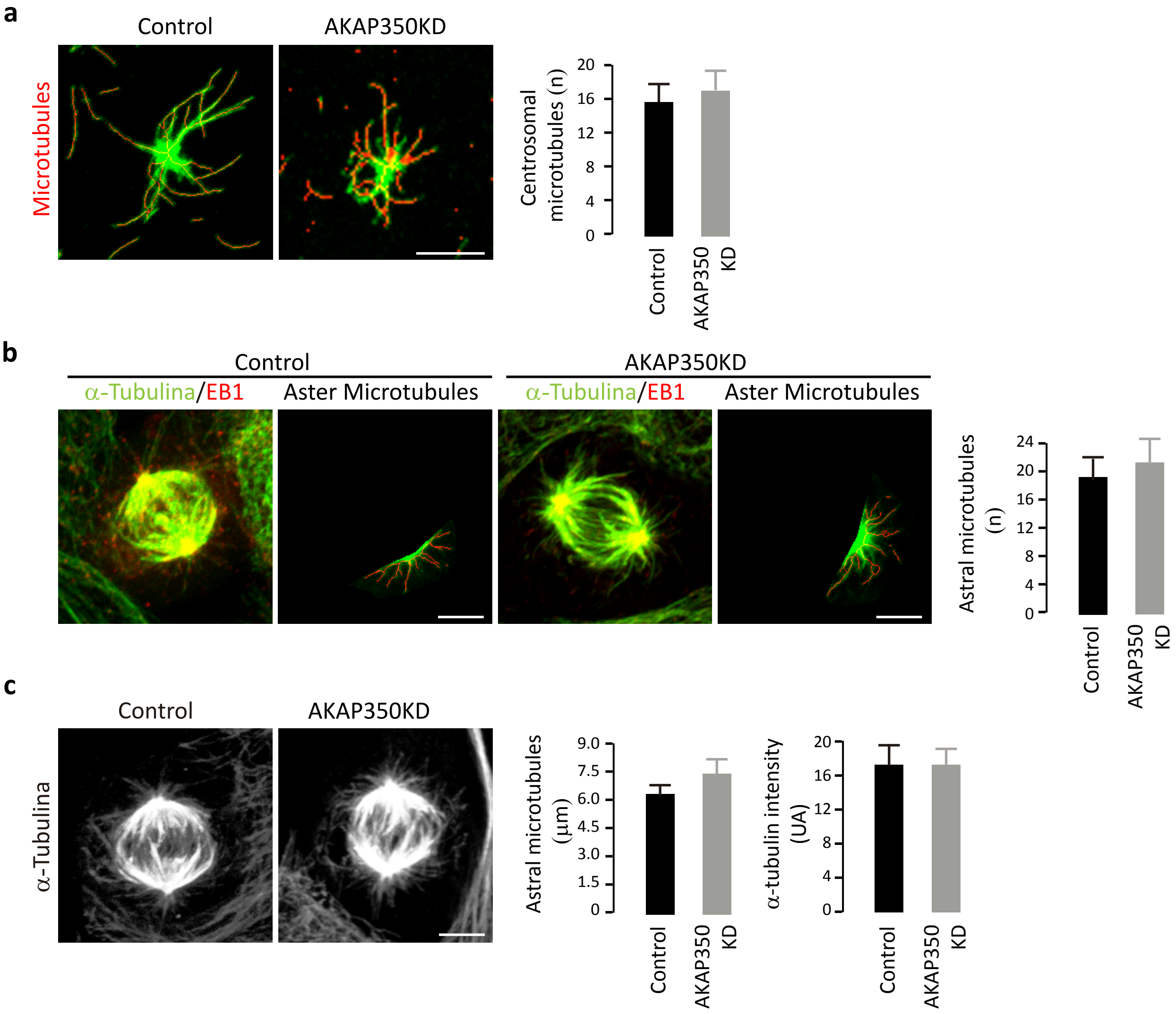

Supplementary Figure 3

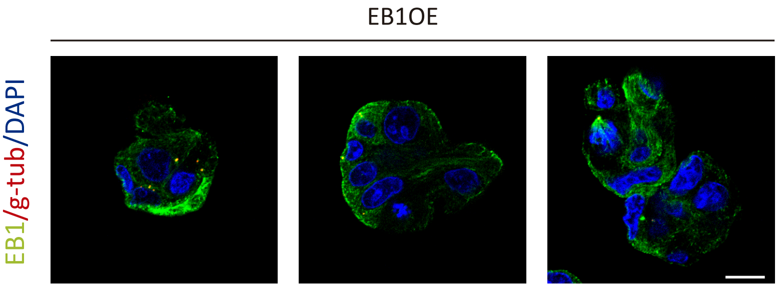

Supplementary Western blots images

a)

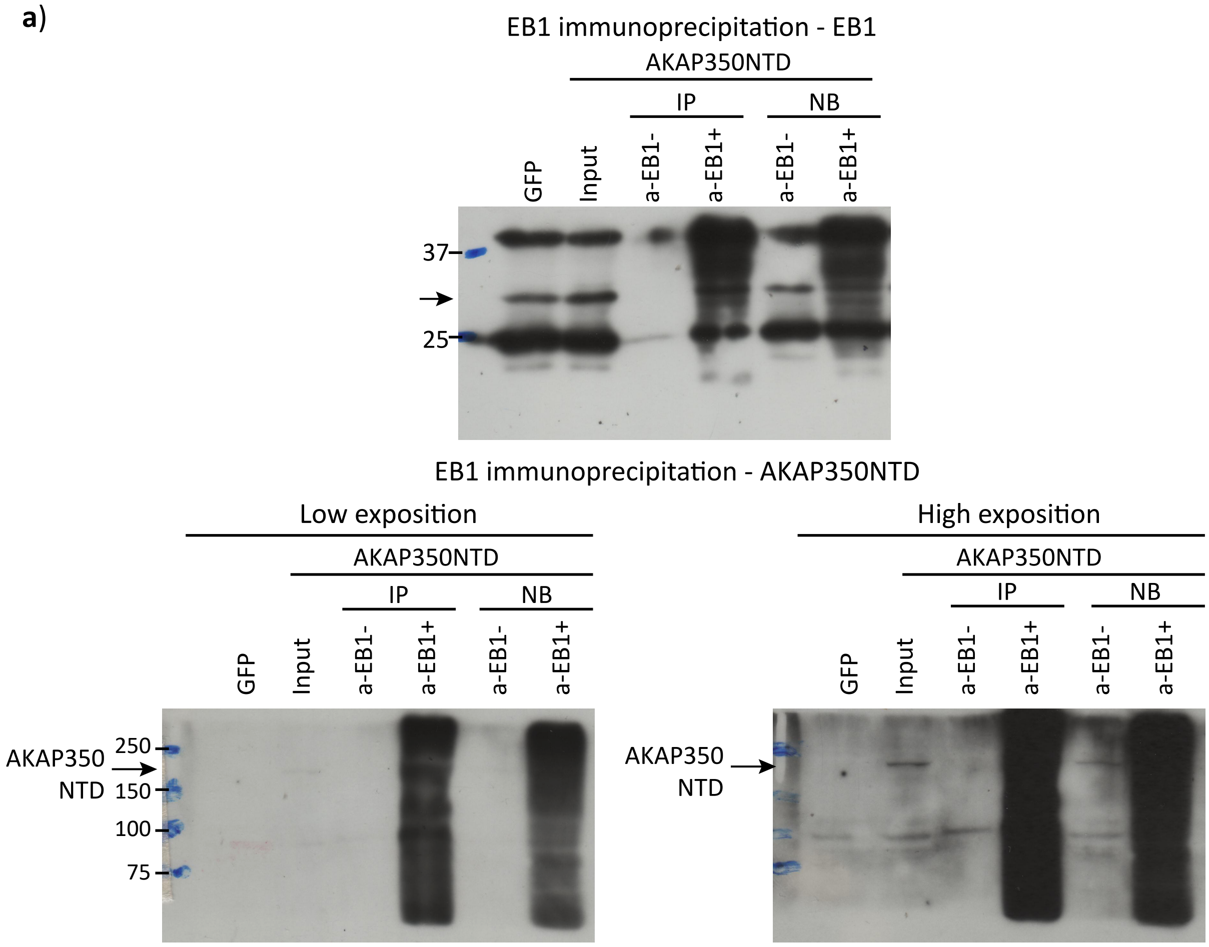

b)

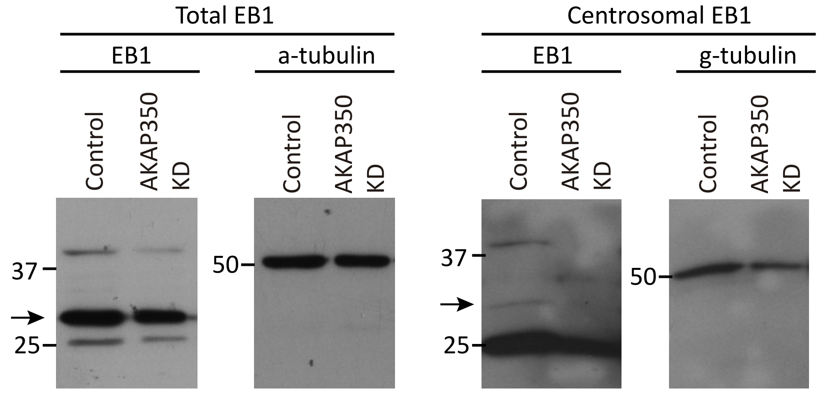

Images show the unedited scans of the western blots corresponding to Figure 3 (A) and Figure 4 (B). (A) AKAP350NTD cell lysates (Input) were incubated with an anti-EB1 antibody (a-EB1+), and subjected to immunoprecipitation using protein A-sepharose beads. Non-bound material (NB) was removed, beads were washed and immunoprecipitates (IP) eluted with sample buffer. The control of immunoprecipitation specificity was prepared by performing the first incubation in the absence of the anti-EB1 antibody (a-EB1-). Samples were analyzed by Western blot using anti-EB1 (top) and anti-GFP (bottom) antibodies. The first lane correspond to control cell lysates expressing GFP. Arrows indicate the immunoreactive band that best fit the predicted molecular weight for EB1 (30 kDa) and AKAP350NTD-GFP fusion protein (157 kDa). (B) Centrosomes enriched subcellular fractions were prepared by differential centrifugation of control or AKAP350KD cell lysates using a Fycoll gradient, as described in Materials and Methods. Cell lyzates and centrosomal enriched fractions were analyzed for EB1 and α-tubulin or γ-tubulin expression by western blot.
